# PrognosiT: Pathway/gene set-based tumour volume prediction using multiple kernel learning

**DOI:** 10.1186/s12859-021-04460-6

**Published:** 2021-11-02

**Authors:** Ayyüce Begüm Bektaş, Mehmet Gönen

**Affiliations:** 1 Graduate School of Sciences and Engineering, Koç University, Istanbul, 34450 Turkey; 2 Department of Industrial Engineering, College of Engineering, Koç University, Istanbul, 34450 Turkey; 3 School of Medicine, Koç University, Istanbul, 34450 Turkey

**Keywords:** Machine learning, Multiple kernel learning, Support vector regression, Gene set analysis, Cancer biology

## Abstract

**Background:**

Identification of molecular mechanisms that determine tumour progression in cancer patients is a prerequisite for developing new disease treatment guidelines. Even though the predictive performance of current machine learning models is promising, extracting significant and meaningful knowledge from the data simultaneously during the learning process is a difficult task considering the high-dimensional and highly correlated nature of genomic datasets. Thus, there is a need for models that not only predict tumour volume from gene expression data of patients but also use prior information coming from pathway/gene sets during the learning process, to distinguish molecular mechanisms which play crucial role in tumour progression and therefore, disease prognosis.

**Results:**

In this study, instead of initially choosing several pathways/gene sets from an available set and training a model on this previously chosen subset of genomic features, we built a novel machine learning algorithm, PrognosiT, that accomplishes both tasks together. We tested our algorithm on thyroid carcinoma patients using gene expression profiles and cancer-specific pathways/gene sets. Predictive performance of our novel multiple kernel learning algorithm (PrognosiT) was comparable or even better than random forest (RF) and support vector regression (SVR). It is also notable that, to predict tumour volume, PrognosiT used gene expression features less than one-tenth of what RF and SVR algorithms used.

**Conclusions:**

PrognosiT was able to obtain comparable or even better predictive performance than SVR and RF. Moreover, we demonstrated that during the learning process, our algorithm managed to extract relevant and meaningful pathway/gene sets information related to the studied cancer type, which provides insights about its progression and aggressiveness. We also compared gene expressions of the selected genes by our algorithm in tumour and normal tissues, and we then discussed up- and down-regulated genes selected by our algorithm while learning, which could be beneficial for determining new biomarkers.

**Supplementary Information:**

The online version contains supplementary material available at 10.1186/s12859-021-04460-6.

## Background

Cancer is one of the most common causes of mortality in our era, and its treatment may extremely be hard to patients, both from a psychological and economical perspective. Many genetic, epigenetic and environmental factors are effective in cancer pathogenesis and for each type of cancer, these factors play different roles. Therefore, determining the molecular mechanisms that are related to driver genes and driver pathways is of great importance in terms of cancer diagnostics, prognostics and treatment. In recent years, projects about large-scale cancer genomics are giving researchers the chance to understand the genomic and epigenomic changes in patients. Thus, associated with the increasing opportunity of analysing genomic characterizations of tumours biopsied from patients, standard machine learning algorithms like random forest (RF) [1] and support vector machines (SVM) [2] have been utilized to make predictions related to cancer. Even though the predictive performance of these machine learning applications is usually good, these applications may not be successful in extracting significant and meaningful knowledge from the data since the genomic data sets are high-dimensional and highly correlated by their nature. For this reason, designing new machine learning algorithms that are capable of selecting meaningful parts of the genomic data sets and use these selected subsets for prediction is necessary.

Tumour volume is considered to be one of the significant prognostic factors for oncological outcome after radiotherapy or chemotherapy [24]. Along with clinical T and N stages, tumour differentiation and circumferential tumour extent, tumour volume has been identified by numerous retrospective cohort studies as potential predictors of pathologic complete response [23]. Rather than TNM staging system, estimating tumour volume from the patient’s genomic data while conjointly identifying the molecular mechanisms that affect tumour progression could be highly useful to foresee cancer aggressiveness. Although TNM staging system has been demonstrated to have prognostic information, different cure rates in the literature have raised concern about the efficiency of the T-classification [13].

Even though a reduction in tumour volume after therapy appears to be indicating a better prognosis than an unchanged or increasing tumour size, this assumption may not be correct in some cases. Tumour size is strongly related to cancer prognosis but dynamics of this relation have not yet been fully understood. Since the underlying biological mechanisms that affect tumour size have not yet been discovered completely, there arises a need to determine new ways to predict cancer prognosis using tumour volume. In other words, there is a need for models that learn to predict tumour volume while determining the important pathways/gene sets that affect the tumour progression and agressiveness at the genomic level [26].

Klement et al. [14] studied SVM-based prediction of local tumour control, but they trained their model on only seven potential input features. While there exists standard statistical tests and models applied to the clinical outcomes of patients; to our knowledge, there is no study of predicting tumour volume using machine learning while simultaneously discovering the hidden molecular mechanisms towards tumour progression using genomic characterizations of the patients as input.

Amongst machine learning algorithms, the kernel-based approaches have been shown to be successful in problems associated with cancer, such as gene essentiality prediction by Gönen et al. [9], due to their capability of handling high-dimensional genomic input data. Using multiple kernel learning framework on multi-omics data, Li et al. built a linear mixed model with adaptive Lasso for phenotype prediction which is capable of selecting predictive regions and predictive layers of the data [15]. As another recent work, Uzunangelov et al. designed a multiple kernel learning framework, a kernel-based stacked learner where kernels are integrated with random forests where each one is built from a specific pathway gene set [25].

For genomic data sets that have relatively low number of training instances, the number of model parameters to be optimized using kernel-based approaches is proportional to the number of training instances (*N*, generally in the order of hundreds); not to the number of training features (*D*, generally in the order of thousands) [20], which is a big computational advantage compared to other machine learning algorithms for this specific problem type.

**Fig. 1:**
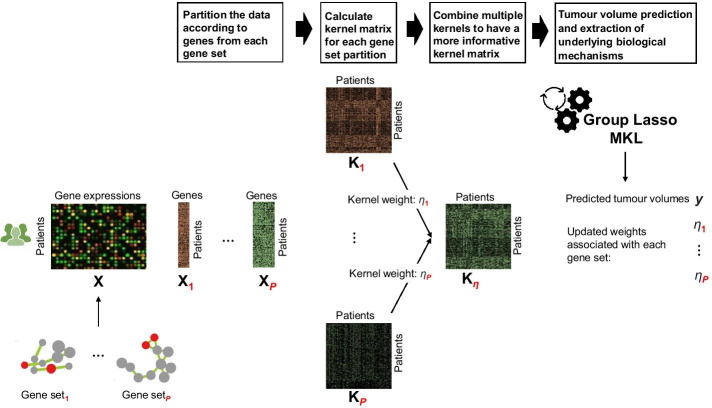
Our proposed MKL algorithm, named PrognosiT, which takes gene expression profiles of patients, denoted as $\mathbf {X}$, tumour volumes of patients, denoted as $\varvec{y}$, and a pathway/gene set collection as its input. Then, it calculates distinct kernel matrices, denoted as $\mathbf {K}_{1}, \dots , \mathbf {K}_{P}$, for input pathways/gene sets on gene expression partitions, denoted as $\mathbf {X}_{1}, \dots , \mathbf {X}_{P}$, formed from the input matrix of gene expression profiles. Followingly, to have a kernel matrix between pairs of patients which is denoted as $\mathbf {K}_{\eta }$ and which carries more information, multiple kernel matrices are combined with a weighted sum. The resulting kernel matrix is later used to learn a function, denoted as *f*, to predict tumour volumes of out-of-sample cancer patients

Most frequently used based learners for multiple kernel learning (MKL) algorithms are SVM and support vector regression (SVR) since they have been proven empirically successful and they are easily applicable as a building block [10]. In this study, we applied MKL algorithm using SVR as base learner on gene sets to discover mechanisms at the molecular level related to tumour initiation and progression (see Fig. 1). We tested the predictive ability of our novel algorithm PrognosiT on the task of predicting tumour volume from genomic data. We also confirmed the relevant pathway/gene set outputs from our algorithm with the existing literature about the studied cancer type (i.e., thyroid carcinoma). Lastly, we compared the tumour and normal tissue gene expressions for the list of genes resulted from our algorithm, which could be beneficial for determining genomic biomarkers for screening.

## Materials

We gathered genomic characterizations and clinical annotation files of over 10 000 cancer patients from 33 cancer cohorts in the Genomics Data Commons (GDC) data portal offered by The Cancer Genome Atlas (TCGA) consortium at https://portal.gdc.cancer.gov. TCGA provided the RNA-Seq measurements of the tumours from 33 cohorts and pre-processed them with a unified pipeline, which facilitated our analysis of tumour gene expression profiles. We downloaded HTSeq-FPKM files of all primary tumours from the most recent data freeze (i.e., Data Release 29-March 31, 2021), which leads to 9911 files in total. Due to the strong elemental and hidden differences at the molecular level, we did not add metastatic tumours to our analysis. We utilized clinical annotation files of the patients to obtain the tumour volume information. We checked the clinical annotation files for tumour dimension information (i.e., tumour length, tumour depth and tumour width) and there were only two cohorts containing this information, namely SARC and THCA. By nature, malignant soft tissue tumours (i.e., sarcomas) have numerous subtypes with different prognoses and therefore, with different molecular mechanisms related to cancer progression. Concordantly, when we checked the histological type information in clinical annotation files, we saw that histological types of cancer tissues were highly different within SARC cohort. Thus, we excluded SARC cohort from further analysis, and used THCA cohort that has 507 patients in total.

We first calculated tumour volume for each tumour by multiplying neoplasm_length, neoplasm_width and neoplasm_depth found in the clinical annotation files. Afterwards, we picked the patients that have both tumour dimension information and gene expression profile. We then discarded patients having their tumour volume non-positive or NA, leading us to 402 primary tumours in THCA cohort. Lastly, to compare the gene expressions between tumour and normal tissues, we used the normal tissue gene expression profiles found in 58 patients.

In addition to having a predictive model for tumour volume, we aimed to discover the molecular processes that play key roles in this volume prediction task. Therefore, we used cancer-specific pathway/gene set collections previously depicted in the literature. Using these collections, we can determine the group of genes having similarities or dependencies in their functionalities.

Using the Molecular Signatures Database (MSigDB), we extracted Hallmark gene sets and Pathway Interaction Database (PID), which are specifically curated for cancer-related research tasks. Hallmark gene set collection contains computationally constructed list of genes that convey a particular biological state or process and shows coherent expression in cancers [16]. PID is a freely available collection of manually curated and peer-reviewed pathways that consists of human molecular signaling and regulatory events and major cellular processes [19]. The Hallmark gene set collection contains 50 gene sets with sizes varying between 32 and 200, whereas the PID collection include 196 pathways with sizes varying between 10 and 137.

## Methods

We approached the problem of predicting the volume of primary tumours at the diagnosis while simultaneously determining the molecular mechanisms that affect tumour progression by applying machine learning algorithms on gene expression profiles extracted from the tumours. For a cohort consisting of *N* patients, the training data set can be represented as $\{(\varvec{x}_i, y_{i})\}_{i = 1}^{N}$, where *N* denotes the number of tumours, $\varvec{x}_i$ denotes the gene expression profile related to tumour *i*, and $y_{i}$ is the volume of tumour *i*.

Aforementioned problem may be formulated using a regression model and can be solved using algorithms for regression such as RF [1] and SVR [6]. With these algorithms, it may be possible to have good predictive performances. However, good prediction performance is not enough to extract insightful information about the mechanisms that play role in tumour progression. It is shown that, for predicting outcome in cancer, size of the training sample set should be at least in the order of thousands [7]. In other words, since gene expression data is highly correlated by its nature, if the training sample set is not in order of thousands, machine learning algorithms might use different subsets of a specific patient cohort to predict and might result with different biomarkers for prediction. Therefore, it would be sensible to utilize our prior knowledge regarding genes, information from pathway/gene sets, and discover mechanisms at the molecular level using this prior information.

### Baseline algorithms

RF algorithm is a combination of weak decision trees, and it uses an ensemble strategy to get more robust classification and regression trees than decision trees algorithm [1]. Because of its data-adaptive structure, RF is appealing for high-dimensional genomic data analysis [3]. We chose RF as a baseline algorithm due to the fact that it is highly used in the studies with genomic input data, and it is capable of handling the noise and the correlation among features [5, 21, 22]. Despite the fact that predictive performance of RF is claimed to be good in certain applications, their capability to extract meaningful knowledge from data is highly inadequate. Additionally, since RF models are generally built by randomly selecting bootstrap samples, their knowledge extraction process may vary remarkably.

SVR is a modified version of SVM algorithm [2] to be used for prediction tasks [6]. Our proposed MKL algorithm uses SVR as the base learner. The mathematical details of the optimization problem used for SVR is

1 $$\begin{aligned} \begin{aligned} \text{ min. }\;\;&\dfrac{1}{2} \varvec{w}^{\top } \varvec{w} + C \sum \limits _{i = 1}^{N} (\xi _{i}^{+} + \xi _{i}^{-}) \\ \text{ w.r.t. }\;\;&\varvec{w} \in {\mathbb {R}}^{D},\;\;\varvec{\xi }^{+} \in {\mathbb {R}}^{N},\;\;\varvec{\xi }^{-} \in {\mathbb {R}}^{N},\;\;b \in {\mathbb {R}} \\ \text{ s.t. }\;\;&\epsilon + \xi _{i}^{+} \ge y_{i} - \varvec{w}^{\top } \varvec{x}_{i} - b \;\;\;\;\;\;\forall i \\&\epsilon + \xi _{i}^{-} \ge \varvec{w}^{\top } \varvec{x}_{i} + b - y_{i} \;\;\;\;\;\;\forall i \\&\xi _{i}^{+} \ge 0\;\;\;\;\;\;\forall i\\&\xi _{i}^{-} \ge 0\;\;\;\;\;\;\forall i, \end{aligned} \end{aligned}$$

where $\varvec{w}$ is the feature weight vector, *C* is the non-negative regularization parameter, $\varvec{\xi }^{+}$ and $\varvec{\xi }^{-}$ are the sets of slack variables, *D* is the number of input features (number of genes in gene expression profiles), *b* is the intercept parameter, and $\epsilon$ is the non-negative tube width parameter.

Solving the dual of the above optimization problem would decrease the number of decision variables and thereby, we could integrate kernel functions in the problem formulation, to be able to model non-linear problems. The corresponding dual optimization problem is

2 $$\begin{aligned} \begin{aligned} \text{ min. }\;\;&-\sum \limits _{i = 1}^{N} y_{i} (\alpha _{i}^{+} - \alpha _{i}^{-}) + \epsilon \sum \limits _{i = 1}^{N} (\alpha _{i}^{+} + \alpha _{i}^{-}) \\ {}&+ \dfrac{1}{2} \sum \limits _{i = 1}^{N} \sum \limits _{j = 1}^{N} (\alpha _{i}^{+} - \alpha _{i}^{-}) (\alpha _{j}^{+} - \alpha _{j}^{-}) \varvec{x}_{i}^{\top } \varvec{x}_{j} \\ \text{ w.r.t. }\;\;&\varvec{\alpha }^{+} \in {\mathbb {R}}^{N},\;\;\varvec{\alpha }^{-} \in {\mathbb {R}}^{N} \\ \text{ s.t. }\;\;&\sum \limits _{i = 1}^{N} (\alpha _{i}^{+} - \alpha _{i}^{-})= 0 \\&C \ge \alpha _{i}^{+} \ge 0\;\;\;\;\;\;\forall i\\&C \ge \alpha _{i}^{-} \ge 0\;\;\;\;\;\;\forall i. \end{aligned} \end{aligned}$$

In the dual optimization problem, there are 2*N* decision variables instead of $(D+2N+1)$, which is the number of decision variables in the primal problem. To build non-linear models, we can add the kernel function to the dual formulation by replacing $\varvec{x}_{i}^{\top } \varvec{x}_{j}$ term with $k(\varvec{x}_i, \varvec{x}_j)$, where we encode the similarities between pairs of data points. This kernel function is usually selected with a model selection approach by trying several alternatives.

### Derivation of dual optimization problem for support vector regression

The Langrangian function corresponding to the primal optimization problem shown in Equation (1) is calculated as

$$\begin{aligned} {\mathcal {L}}= & {} \dfrac{1}{2} \varvec{w}^{\top } \varvec{w} + C \sum \limits _{i = 1}^{N} (\xi _{i}^{+} \xi _{i}^{-}) - \sum \limits _{i = 1}^{N} \alpha _{i}^{+} (\epsilon + \xi _{i}^{+} - y_{i} + \varvec{w}^{\top } \varvec{x}_{i} + b) \\&- \sum \limits _{i = 1}^{N} \beta _{i}^{+} \xi _{i}^{+} - \sum \limits _{i = 1}^{N} \alpha _{i}^{-} (\epsilon + \xi _{i}^{-} - \varvec{w}^{\top } \varvec{x}_{i} - b + y_{i}) - \sum \limits _{i = 1}^{N} \beta _{i}^{-} \xi _{i}^{-}, \end{aligned}$$

after that we take the derivative of the Lagrangian function with respect to all the decision variables of the primal optimization problem, we get

$$\begin{aligned} \dfrac{\partial {\mathcal {L}}}{\partial \varvec{w}}&= 0 \Rightarrow \varvec{w} = \sum \limits _{i = 1}^{N} (\alpha _{i}^{+} - \alpha _{i}^{-}) \varvec{x}_{i} \\ \dfrac{\partial {\mathcal {L}}}{\partial b}&= 0 \Rightarrow \sum \limits _{i = 1}^{N} (\alpha _{i}^{+} - \alpha _{i}^{-}) = 0 \\ \dfrac{\partial {\mathcal {L}}}{\partial \xi _{i}^{+}}&= 0 \Rightarrow C = \alpha _{i}^{+} + \beta _{i}^{+} \;\;\;\;\;\;\forall i \\ \dfrac{\partial {\mathcal {L}}}{\partial \xi _{i}^{-}}&= 0 \Rightarrow C = \alpha _{i}^{-} + \beta _{i}^{-} \;\;\;\;\;\;\forall i. \end{aligned}$$

Lastly, by plugging the derivation results back into the Lagrangian function we obtain the dual optimization problem shown in Equation (2).

### Our proposed algorithm

The predictive ability of machine learning algorithms that uses “kernel trick” to capture patterns between pairs of data points is quite dependent on the selected kernel function. The standard practice for selecting a kernel function consists of first comparing the performances of several candidate kernel functions with the aid of a cross-validation technique on the training data and then selecting the kernel function that performs best on the training set to make predictions on the test set. Nevertheless, usage of a single kernel function might not be sufficient for handling the complexity of the studied machine learning problem, but a combination of different kernels might give better predictive results than a single one. Moreover, there exist many algorithms that combine multiple kernels to capture the similarity between pairs of data points. Different kernels in MKL may correspond to using different measures of similarity or they may be using information coming from different sources (i.e., different feature representations or different feature subsets) [10]. For instance, MKL algorithms might combine kernel functions that have different complexities (e.g., linear, polynomial or Gaussian) defined on the same input representation or they might combine kernels prepared from different sources of input data (i.e., multiview learning; data fusion from multiple feature sets). To be used in cancer research, we can train algorithms using the same set of tumours in different representations such as gene expression, copy number or methylation profiles.

In this study, one of our purposes was to discover biological mechanisms that define tumour prognosis. To that end, we propose a modified version of SVR using multiple kernel learning on pathways/gene sets (PrognosiT). We first form a kernel matrix for each pathway/gene set and then we combine these kernel matrices using an MKL algorithm. We assume that we are given *P* kernel functions instead of a single one for PrognosiT algorithm, where we calculate a weighted sum of these kernel functions. In other words, we get a convex combination of these kernel functions (i.e., sum of non-negative kernel weights is set equal to one).

To integrate MKL into the SVR model, the dual of the support vector optimization model can be used as an inner problem within the following outer optimization model:

3 $$\begin{aligned} \begin{aligned} \text{ min. }\;\;&J(\varvec{\eta }) \\ \text{ w.r.t. }\;\;&\varvec{\eta } \in {\mathbb {R}}^{P} \\ \text{ s.t. }\;\;&\sum \limits _{m = 1}^{P} \eta _{m} = 1 \\&\eta _{m} \ge 0\;\;\;\;\;\;\forall m, \end{aligned} \end{aligned}$$

where $\varvec{\eta }$ represents the kernel weights, and $J(\varvec{\eta })$ is the optimization problem depicted in Equation (2) with a modified objective function, that replaces $k(\varvec{x}_{i}, \varvec{x}_{j}) = \varvec{x}_{i}^{\top } \varvec{x}_{j}$ term with $\sum \nolimits _{m = 1}^{P} \eta _{m} k_{m}(\varvec{x}_{i}, \varvec{x}_{j})$. It should be noted that the equality constraint in Equation (3), which is otherwise known as the unit simplex constraint, represents enforcing $\ell _{1}$-norm on the kernel weights and leads us to have a sparse solution. The optimization problem in Equation (3) is convex with respect to $\varvec{\eta }$, and the optimization problem in Equation (2) is convex with respect to $\{\varvec{\alpha }^{+},\varvec{\alpha }^{-}\}$, but the nested optimization problem is not convex with respect to $\varvec{\eta }$ and $\{\varvec{\alpha }^{+},\varvec{\alpha }^{-}\}$. Since we cannot solve this nested optimization problem globally, we inspire from group Lasso MKL algorithm that was initially constructed for binary classification tasks [27]. We initiate the algorithm by setting all kernel weights equal to each other. Since the summation of all kernel weights should be one, each kernel weight is 1/*P* at the initialization. We then solve the inner optimization problem (i.e., a standard SVR model) at each iteration *t* by utilizing the current kernel weights $\varvec{\eta }^{(t)}$ to obtain the support vector coefficients $\{\varvec{\alpha }^{+(t)},\varvec{\alpha }^{-(t)}\}$. We then calculate the kernel weights at the next iteration using the following update equation:

$$\begin{aligned} \eta _{m}^{(t + 1)}&= \dfrac{\eta _{m}^{(t)} \sqrt{\sum \limits _{i = 1}^{N} \sum \limits _{j = 1}^{N} \alpha _{i}^{(t)} \alpha _{j}^{(t)} k_{m}(\varvec{x}_{i}, \varvec{x}_{j})}}{\sum \limits _{o = 1}^{P} \eta _{o}^{(t)} \sqrt{\sum \limits _{i = 1}^{N} \sum \limits _{j = 1}^{N} \alpha _{i}^{(t)} \alpha _{j}^{(t)} k_{o}(\varvec{x}_{i}, \varvec{x}_{j})}} \;\;\;\;\;\;\forall m, \end{aligned}$$

where $\alpha _{i}^{(t)} = (\alpha _{i}^{+(t)} - \alpha _{i}^{-(t)})$, and the superscripts (*t*) and $(t + 1)$ depicts the current and next iterations, respectively. This is an iterative solution methodology and is demonstrated to converge for binary classification problems [27]. In each iteration, we can solve the inner optimization problem to optimality since it is a standard SVR formulation when we fix the kernel weights $\varvec{\eta }$. We can also solve the outer optimization problem to optimality when we fix the sample weights $\{\varvec{\alpha }^{+}, \varvec{\alpha }^{-}\}$. These two steps are monotonically decreasing the objective function, leading to convergence.

### Kernel selection

When our algorithm converges, we can note the final $\varvec{\eta }$ values to identify which kernels are included (i.e., non-zero $\eta _{m}$ values) in the final model. We perform kernel selection in a supervised manner by conjointly learning regression coefficients and kernel weights. Thanks to the $\ell _{1}$-norm on the kernel weights, we obtain sparse kernel weights, leading to eliminating irrelevant kernel from the combination. Additionally, we can compare the significance of pathways/gene sets by comparing their kernel weights, which could give us valuable information about biological processes towards tumour progression.

## Results and discussion

For the purpose of testing our PrognosiT algorithm, we performed computational experiments on TCGA thyroid carcinoma data set, which contains the volume information of tumours. We compared PrognosiT against two baseline algorithms that are widely used for genomic data analysis, namely, RF and SVR.

### Experimental settings

We split the data set into two parts, 80% of the tumours formed the training set and the remaining 20% of the tumours formed the test set. We normalized each feature in the training set to have zero mean and unit standard deviation. For the test set, we normalized each feature with the mean and standard deviation calculated on the original training set. We applied cube root transformation to the tumour volume values since it is the multiplication of three dimensions. We performed 100 replications of our analysis to have more robust results, and we reported the results of these 100 replications. The hyper-parameters for RF (i.e., number of trees to grow), SVR (i.e., regularization parameter C and tube width multiplier) and MKL (i.e., regularization parameter C and tube width multiplier) were selected by utilizing a four-fold cross-validation strategy on the training set.

We used randomForestSRC R package version 2.9.3 [12] for RF experiments. We chose the number of trees to grow, ntree, from the set $\{500,1000,\dots ,2500\}$ by the aforementioned cross-validation approach.

For SVR and our proposed MKL algorithm PrognosiT, we built our own implementations in R, which use CPLEX version 12.6.3 for solving quadratic optimization problems [11]. We used Gaussian kernel in our algorithm to form a similarity measure between gene expression profiles of primary thyroid tumours. The Gaussian kernel is:

$$\begin{aligned} k_{{\mathcal {G}}}(\varvec{x}_{i}, \varvec{x}_{j})&= \exp \left( -\dfrac{(\varvec{x}_{i} - \varvec{x}_{j})^{\top } (\varvec{x}_{i} - \varvec{x}_{j})}{2\sigma ^{2}}\right) , \end{aligned}$$

where $\sigma$ is the kernel width parameter, and we set it to the mean pairwise Euclidean distances between training samples. For both algorithms, the tube width multiplier parameter, which is to be multiplied by the standard deviation of the current training samples to form the tube width, is chosen from the set $\{0,0.25,0.50,\dots ,2\}$, whereas the regularization parameter *C* is chosen from the set $\{10^{-3}, 10^{-2}, \dots , 10^{+3}\}$ using the previously described four-fold inner cross-validation strategy.

In our implementation, we chose Gaussian kernel to discover the highly non-linear dependency between the tumour progression and gene expression profiles. The Gaussian kernel function was validated previously in two studies as trustworthy to be used with high-dimensional genomic data [4, 9]. We calculated the Gaussian kernel matrices on subsets of tumour gene expression profiles by examining the pathway and gene set content and selecting the corresponding kernel widths. For PrognosiT algorithm, knowing that the algorithm converges in the order of tens of iterations, we performed 200 iterations to guarantee the convergence.

### Performance metric

We used a form of normalized root mean squared error (i.e., NRMSE) for comparison of prediction performances of the three algorithms, namely, RF, SVR and our proposed MKL algorithm PrognosiT. NRMSE can be calculated as

$$\begin{aligned} \text {NRMSE} = \sqrt{\dfrac{(\varvec{y} - \widehat{\varvec{y}})^{\top } (\varvec{y} - \widehat{\varvec{y}})}{(\varvec{y} - \varvec{1} y_{.})^{\top } (\varvec{y} - \varvec{1} y_{.})}}, \end{aligned}$$

where $\varvec{y}$ and $\widehat{\varvec{y}}$ stand for the vectors of observed and predicted tumour volumes, respectively, and $y_{.}$ denotes the mean of $\varvec{y}$. Note that smaller NRMSE values correspond to better predictive performance, and if the value is less than one, it means that the model is capable of learning from the data set.

### Predictive performance of PrognosiT

We compared three machine learning algorithms in our experiments: random forest (denoted as RF), support vector regression (denoted as SVR) and our proposed algorithm PrognosiT that integrates multiple kernel learning (denoted as MKL). For RF and SVR algorithms, we provided all the available gene expression features (i.e., 19 814 features in total) as the input data. For PrognosiT algorithm, MKL[H] and MKL[P] utilized the Hallmark and PID pathway/gene set collections, respectively.

**Fig. 2:**
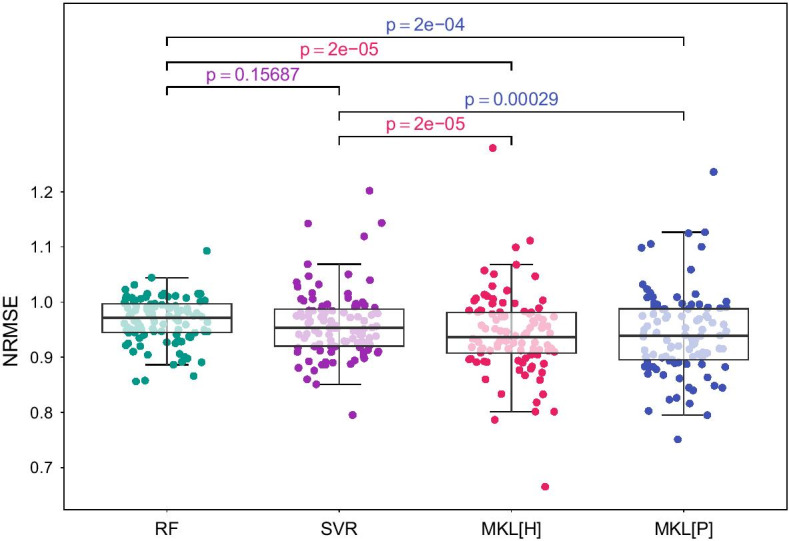
The predictive performance of RF algorithm (RF), SVR algorithm (SVR), PrognosiT algorithm with the Hallmark gene set collection (MKL[H]) and PrognosiT algorithm with the Pathway Interaction Database pathway collection (MKL[P]) on thyroid carcinoma (THCA) data set. Comparison of the NRMSE values resulted from 100 replications for each of the four algorithms is made using the box-and-whisker plots. RF is compared against SVR, MKL[H] and MKL[P] algorithms using the two-tailed paired *t*-test to check whether there is a significant difference between their performances. In addition, SVR is compared against MKL[H] and MKL[P]. The resulting *p*-values are reported. The color of *p*-values matches with the color of the winning algorithm, for each pairwise comparison

Figure 2 displays the predictive performances of RF, SVR, MKL[H] and MKL[P] algorithms on thyroid carcinoma (i.e., THCA) data set for tumour volume prediction problem by using gene expression profiles as the input. The box-and-whisker plots compares the NRMSE values of the four algorithms resulted from 100 random training/test splits. RF algorithm is compared against SVR, MKL[H] and MKL[P] algorithms using a two-tailed paired *t*-test to check whether there is a significant difference between their performances whilst SVR is compared against MKL[H] and MKL[P] algorithms. The NRMSE values of these algorithms resulted from each replication for THCA data set is available in Additional file 2: Table S1.

We observed that SVR algorithm outperformed RF in thyroid carcinoma data set. Due to having a highly non-linear kernel (i.e., the Gaussian kernel) integrated in its implementation, SVR performed better than RF in this prediction task. Our algorithm, PrognosiT, which is an extension of SVR algorithm and integrates our prior knowledge about pathways/gene sets into the machine learning model with a multiple kernel learning formulation, outperformed both RF and SVR.

MKL[H] and MKL[P] algorithms picked significantly fewer gene expression features than RF and SVR algorithms and eliminated uninformative pathways/gene sets from the machine learning model thus, identified relevant pathways/gene sets for tumour volume prediction. RF and SVR algorithms benefited from all available gene expression features (i.e., 19 814 features in total) whereas the average numbers of used gene expression features by MKL[H] and MKL[P] algorithms were respectively 1782 and 797. The exact numbers of gene expression features that are utilized by these algorithms in each replication are available in Additional file 2: Table S2.

### PrognosiT determines informative pathways/gene sets for tumour progression

In addition to comparing the predictive performances of MKL[H] and MKL[P] algorithms to RF and SVR algorithms, we investigated the pathways/gene sets chosen by our proposed PrognosiT algorithm on thyroid carcinoma data set. Additional file 2: Table S3 displays the selection frequencies of 50 gene sets in the Hallmark collection for 100 replications. We assumed that a gene set/pathway was added in the final model in case the corresponding kernel weight was greater than 0.01. The exact kernel weights assigned to 50 gene sets in 100 replications for thyroid carcinoma cohort is displayed in Additional file 2: Table S4. Moreover, we also reported the selection frequencies of 196 pathways in the PID collection for 100 replications in Additional file 2: Table S5 and the exact kernel weights assigned to these pathways in 100 replications in Additional file 2: Table S6. The selection frequencies in MKL[H] algorithm averaged to 28.5 gene sets, whereas those of MKL[P] algorithm averaged to 13.4 pathways.

We checked the column sums of the selection frequencies of the pathways/gene sets for the Hallmark and PID collections shown in Additional file 2: Tables S3 and S5 to discover informative and uninformative gene sets for tumour volume prediction and showed them in Additional file 1: Fig. S1. In the final model, MKL[H] algorithm chose HYPOXIA gene set in 99 replications over 100. Hypoxia is known as one of the most important signatures of solid tumours and is related to radiotherapy and chemotherapy resistance, which leads to poor clinical prognosis [18]. We know that thyroid cancer is highly an ERK-driven malignity and mutations that activate the RAS/ERK mitogenic signaling pathway are responsible for up to 70% of thyroid carcinomas [28]. Thus, having high selection frequencies for KRAS signaling gene sets in the final model shows that our predictive model is in agreement with the literature. Another gene set that our model selected frequently over 100 replications is GLYCOLYSIS. It is known that a near-universal property of primary and metastatic cancers is up-regulation of glycolysis, leading to increased glucose consumption [8].

One other highly selected pathway that attracted our attention in our final model is P53_PATHWAY. It is well known that there exists a complex network among p53 family members and interactions of these members with other elements accelerates thyroid cancer progression [17]. High selection frequency over 100 replications by our final model of ANGIOGENESIS, INFLAMMATORY_RESPONSE and EPITHELIAL_MESENCHYMAL_TRANSITION gene sets, which are highly associated with tumour progression and rapid changes in cellular phenotype, and frequent selection of the metabolism-related gene sets such as PANCREAS_BETA_CELLS, XENOBIOTIC_METABOLISM, FATTY_ACID_METABOLISM, BILE_ACID_METABOLISM show that the results of our proposed algorithm are consistent with the existing knowledge and may contribute to the discovery of unknown mechanisms towards tumour progression.

**Fig. 3:**
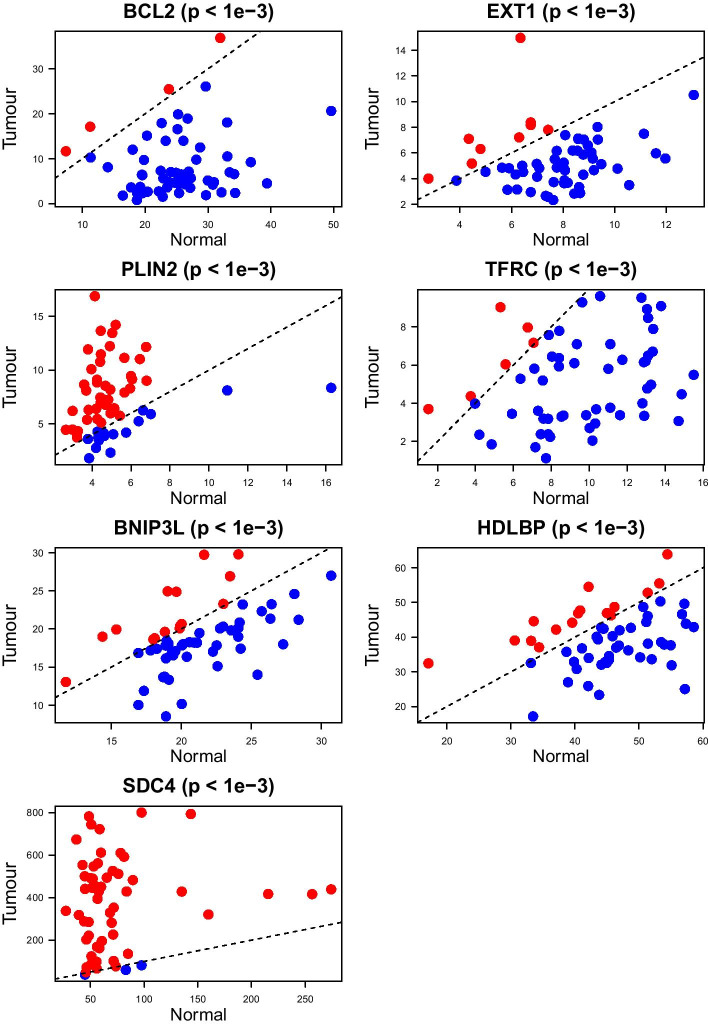
Comparison of mRNA gene expression levels of several genes resulted by our algorithms MKL[H] and MKL[P] on thyroid carcinoma data set. Corresponding gene up- and down-regulations can be understood by scatter plot visualizations of Tumour and Normal tissues. Red points refers to overexpression of the specified gene in tumour whereas blue points refer to underexpression in tumour. The *p*-values are resulted from paired Wilcoxon test that we used to determine whether there is a significant difference between the gene expressions of Tumour and Normal tissues

### PrognosiT reveals significantly up- and down-regulated genes between tumour and normal tissues

After comparing predictive performances and analysing highly selected pathways/gene sets to check the biological mechanisms that lead to tumour progression, we also analysed the genes that have been selected frequently by our final model over 100 replications. We noted the genes that are selected in every replication separately by our MKL[H] and MKL[P] algorithms. There were 137 genes resulted from MKL[H] algorithm and 37 genes resulted from MKL[P] algorithm. We then checked whether these genes are significantly up- or down-regulated during the tumour progression by comparing the gene expressions of tumour tissues to the gene expressions of normal tissues collected from the same patients. There were 58 cancer patients that had both tumour and normal tissue information. We performed Wilcoxon test, which is a non-parametric statistical test that compares two paired groups, to determine whether there is a significant difference (i.e., *p*-value $< 0.05$) between the gene expressions coming from tumour and normal tissues. As a result, there were 113 genes that are significantly up- or down-regulated resulted by MKL[H] and 32 genes resulted by MKL[P].

We showed the scatter plot visualisations of some of the significantly differed expressions of the genes resulted from our MKL[H] and MKL[P] algorithms (see Fig. 3). We checked whether these genes are used as prognostic factor for thyroid cancer evaluation from The Human Protein Atlas website at https://www.proteinatlas.org. The displayed genes in Fig. 3 are already in use as prognostic factor for thyroid cancer. This situation contributes to the possibility that the remaining genes resulted from our algorithm to predict tumour volume might be considered as prognostic factors in the future as well.

## Conclusions

Predicting tumour volume while discovering the underlying molecular mechanisms towards tumour progression using genomic characterizations of cancer patients is critical to foresee the disease prognosis and to be able to develop new therapeutic strategies. This study was designed due to scarcity of integrated computational methods that perform tumour volume prediction and knowledge extraction simultaneously on genomic data, to get insightful information related to cancer progression. Instead of solving these problems separately (i.e., tumour volume prediction and knowledge extraction at separate times), using our integrated approach, we are able to gather robust knowledge about the molecular mechanisms that are related to tumour progression.

We tested our proposed algorithm PrognosiT on thyroid carcinoma cohort (i.e., THCA) from TCGA using two pathway/gene set collections, which are curated specifically for cancer, namely, Hallmark gene set collection [16] and PID pathway collection [19], as prior knowledge source. The predictive performance results we obtained showed that PrognosiT performed comparable or even statistically significantly better than RF [1] and SVR [6] (Fig. 2; Additional file 2: Table S1), which are two standard baseline machine learning algorithms used for prediction from genomic data. The power of our method comes from the fact that the number of used gene expression features was significantly fewer (i.e., less than one-tenth) in PrognosiT while having comparable or even better predictive performance results while conjointly extracting the relevant pathways to predict the tumour volume.

To show the biological relevance of the results of our algorithm, we provided the selection frequencies of pathways/gene sets for THCA data set (Additional file 1: Fig. S1). We also showed the unique genes that are selected in every replication of our algorithm and checked their gene expression levels between tumour and normal tissues. Among these genes, we displayed several statistically significantly up- or down-regulated ones (Fig. 3; Additional file 1: Figs. S2 and S3). We saw that frequently selected pathways/gene sets and unique genes in THCA cohort are supported by the existing literature, and some of the genes that are resulted from our algorithm are already in use as prognostic factors for thyroid carcinoma.

Even though the regression problem in this study is about to predict tumour volumes of cancer patients utilizing their gene expression profiles, it is possible to easily adapt the used computational framework to other disease types, other phenotypes and other prior knowledge sources with slight modifications. However, since there exist different underlying mechanisms related to different diseases, the prior knowledge source should give us insightful information about the studied prediction task. Thus, the compatibility of pathway/gene set collection with the studied prediction problem should be the priority to get good predictive performance using PrognosiT.

## Supplementary Information

**Additional file 1.** Supplementary figures.

**Additional file 2.** Supplementary tables.

## Data Availability

Our implementations of support vector regression and PrognosiT for tumour volume prediction in R can be found at https://github.com/begumbektas/prognosit together with the scripts that replicate the reported experiments.
