# Supplementary material for: PrognosiT: Pathway/gene set-based tumour volume prediction using multiple kernel learning
Source: BMC Bioinformatics. 2021 Nov 2;22:537. doi: 10.1186/s12859-021-04460-6 (PMC8561914; doi:10.1186/s12859-021-04460-6)
Supplement: Supplementary file 1 — Additional file 1. Supplementary figures. [file 12859_2021_4460_MOESM1_ESM.pdf]

PrognosiT: Pathway/gene set-based tumour volume prediction using multiple kernel learning — Supplementary figures —

Ayyüce Begüm Bektaş[1] and Mehmet Gönen[2,3]

[1]Graduate School of Science and Engineering, Koç University, İstanbul 34450, Turkey
[2]Department of Industrial Engineering, College of Engineering, Koç University, İstanbul 34450, Turkey
[3]School of Medicine, Koç University, İstanbul 34450, Turkey

HALLMARK_HYPOXIA	99	PID_CONE_PATHWAY	98
HALLMARK_KRAS_SIGNALING_DN	99	PID_HIF1_TFPATHWAY	98
HALLMARK_SPERMATOGENESIS	96	PID_NFAT_TFPATHWAY	88
HALLMARK_PANCREAS_BETA_CELLS	95	PID_ARF6_PATHWAY	85
HALLMARK_INFLAMMATORY_RESPONSE	86	PID_HNF3A_PATHWAY	85
HALLMARK_XENOBIOTIC_METABOLISM	85	PID_FRA_PATHWAY	84
HALLMARK_FATTY_ACID_METABOLISM	75	PID_ERBB_NETWORK_PATHWAY	82
HALLMARK_NOTCH_SIGNALING	73	PID_HNF3B_PATHWAY	81
HALLMARK_KRAS_SIGNALING_UP	62	PID_WNT_SIGNALING_PATHWAY	77
HALLMARK_ANGIOGENESIS	54	PID_RHODOPSIN_PATHWAY	77
HALLMARK_P53_PATHWAY	41	PID_UPA_UPAR_PATHWAY	74
HALLMARK_MYOGENESIS	39	PID_TCR_CALCIUM_PATHWAY	66
HALLMARK_GLYCOLYSIS	37	PID_FANCONI_PATHWAY	63
HALLMARK_BILE_ACID_METABOLISM	34	PID_LIS1_PATHWAY	63
HALLMARK_HEDGEHOG_SIGNALING	33	PID_HES_HEY_PATHWAY	60
HALLMARK_EPITHELIAL_MESENCHYMAL_TRANSITION	33	PID_IL23_PATHWAY	57
HALLMARK_COAGULATION	32	PID_ERB_GENOMIC_PATHWAY	53
HALLMARK_CHOLESTEROL_HOMEOSTASIS	30	PID_VEGF_VEGFR_PATHWAY	49
HALLMARK_G2M_CHECKPOINT	30	PID_ATR_PATHWAY	46
HALLMARK_E2F_TARGETS	30	PID_ALPHA_SYNUCLEIN_PATHWAY	46
HALLMARK_APICAL_JUNCTION	26	PID_SYNDECAN_4_PATHWAY	44
HALLMARK_APOPTOSIS	23	PID_FOXM1_PATHWAY	43
HALLMARK_ESTROGEN_RESPONSE_LATE	22	PID_BETA_CATENIN_NUC_PATHWAY	43
HALLMARK_MYC_TARGETS_V2	19	PID_EPHB_FWD_PATHWAY	39
HALLMARK_TNFA_SIGNALING_VIA_NFKB	18	PID_CMYB_PATHWAY	39
HALLMARK_ADIPOGENESIS	18	PID_REG_GR_PATHWAY	37
HALLMARK_PEROXISOME	18	PID_PLK1_PATHWAY	37
HALLMARK_WNT_BETA_CATENIN_SIGNALING	13	PID_CD8_TCR_DOWNSTREAM_PATHWAY	37
HALLMARK_DNA_REPAIR	12	PID_TRAIL_PATHWAY	34
HALLMARK_COMPLEMENT	12	PID_CDC42_PATHWAY	33
HALLMARK_MYC_TARGETS_V1	12	PID_LKB1_PATHWAY	32
HALLMARK_INTERFERON_GAMMA_RESPONSE	10	PID_P53_DOWNSTREAM_PATHWAY	30
HALLMARK_IL2_STAT5_SIGNALING	10	PID_GLYPICAN_1PATHWAY	29
HALLMARK_ANDROGEN_RESPONSE	9	PID_SYNDECAN_3_PATHWAY	28
HALLMARK_HEME_METABOLISM	7	PID_FGF_PATHWAY	28
HALLMARK_TGF_BETA_SIGNALING	6	PID_SYNDECAN_1_PATHWAY	26
HALLMARK_INTERFERON_ALPHA_RESPONSE	6	PID_IL3_PATHWAY	25
HALLMARK_REACTIVE_OXIGEN_SPECIES_PATHWAY	6	PID_NEPHRIN_NEPH1_PATHWAY	24
HALLMARK_ESTROGEN_RESPONSE_EARLY	5	PID_DELTA_NP63_PATHWAY	24
HALLMARK_APICAL_SURFACE	3	PID_RAS_PATHWAY	24
HALLMARK_UV_RESPONSE_UP	3	PID_AP1_PATHWAY	22
HALLMARK_IL6_JAK_STAT3_SIGNALING	2	PID_MYC_REPRESS_PATHWAY	22
HALLMARK_PI3K_AKT_MTOR_SIGNALING	2	PID_REELIN_PATHWAY	21
HALLMARK_UV_RESPONSE_DN	2	PID_PRL_SIGNALING_EVENTS_PATHWAY	20
HALLMARK_MITOTIC_SPINDLE	0	PID_AURORA_B_PATHWAY	20
HALLMARK_PROTEIN_SECRETION	0	PID_ARF_3PATHWAY	20
HALLMARK_UNFOLDED_PROTEIN_RESPONSE	0	PID_INSULIN_GLUCOSE_PATHWAY	20
HALLMARK_MTORC1_SIGNALING	0	PID_PS1_PATHWAY	19
HALLMARK_OXIDATIVE_PHOSPHORYLATION	0	PID_P38_MK2_PATHWAY	17
HALLMARK_ALLOGRAFT_REJECTION	0	PID_AVB3_OPN_PATHWAY	16

Figure S1: The selection frequencies of gene sets in the `Hallmark` collection over 100 replications resulted from PrognosiT algorithm and the selection frequencies of top 50 pathways in the `PID` collection over 100 replications resulted from PrognosiT algorithm.

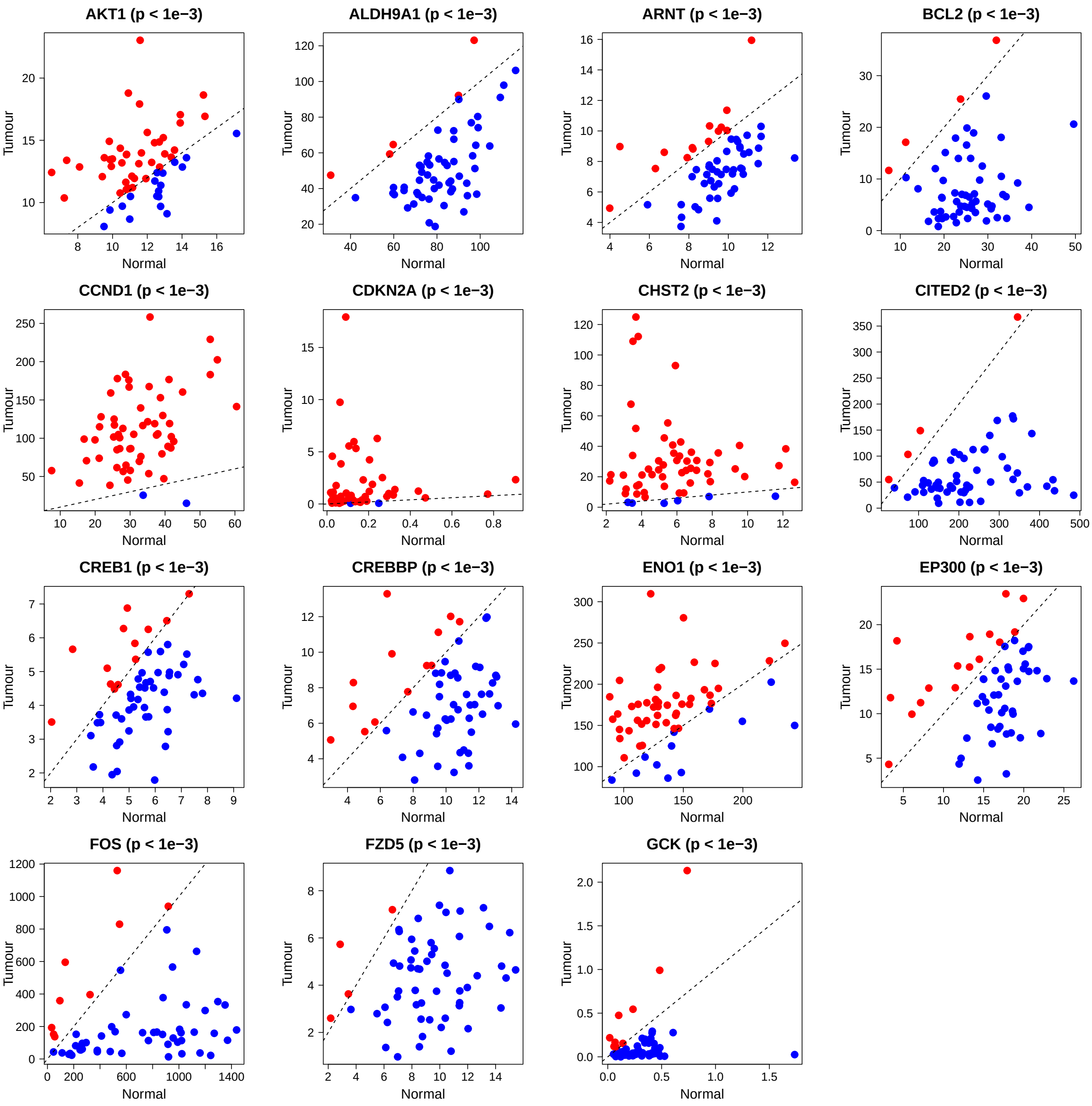

Figure S2: Statistically significantly up- or down-regulated genes during tumor progression selected by PrognosiT algorithm on thyroid carcinoma (i.e., `THCA`) data set over 100 replications. We performed paired Wilcoxon test to determine whether there is a significant difference (i.e., p-value < 0.05) between the gene expressions coming from tumor and normal tissues.

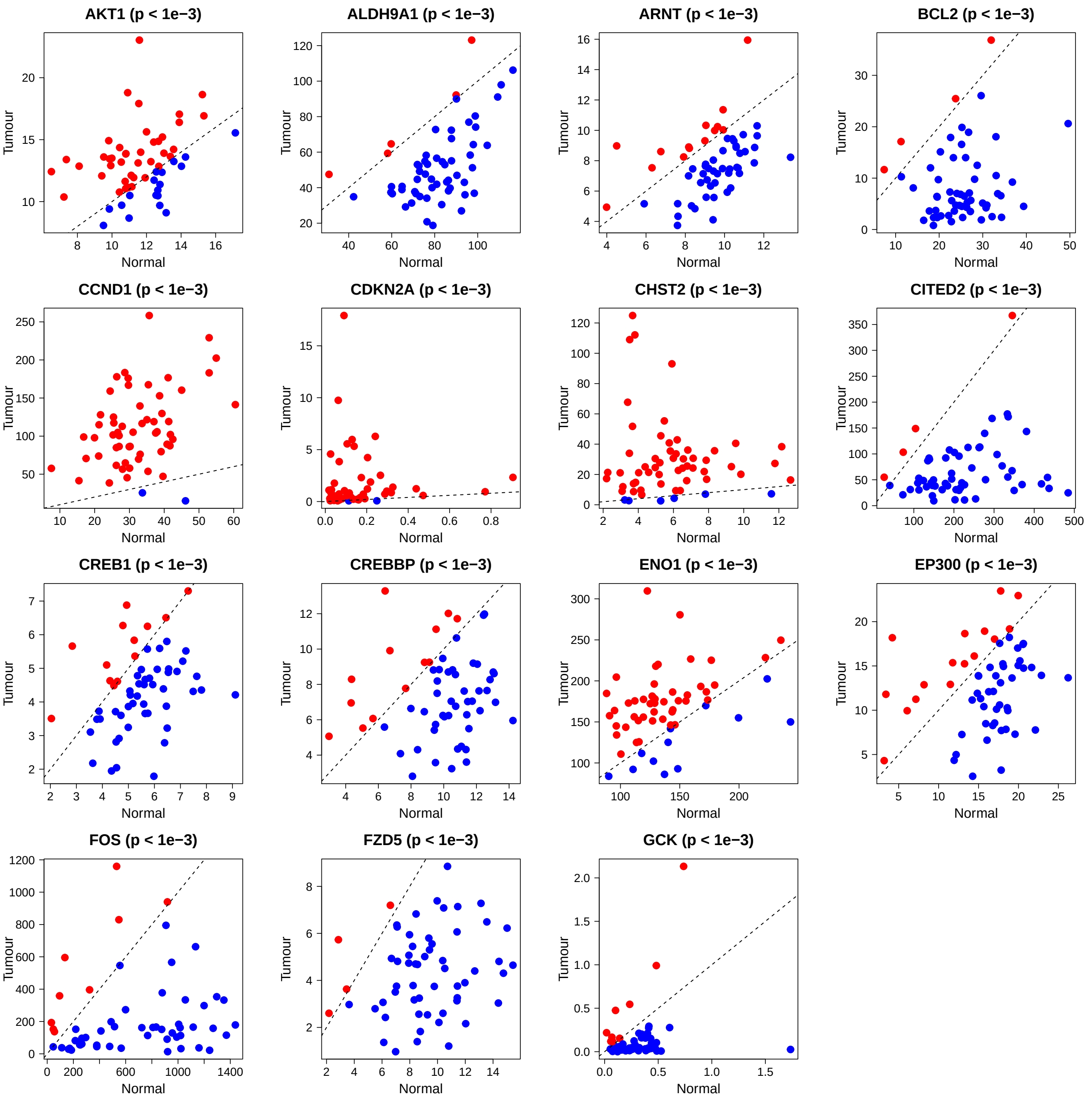

Figure S3: Statistically significantly up- or down-regulated genes during tumor progression selected by PrognosiT algorithm on thyroid carcinoma (i.e., `THCA`) data set over 100 replications. We performed paired Wilcoxon test to determine whether there is a significant difference (i.e., p-value < 0.05) between the gene expressions coming from tumor and normal tissues.
